# Immunoglobulin light chain (IgL) genes in zebrafish: Genomic configurations and inversional rearrangements between (V_L_–J_L_–C_L_) gene clusters

**DOI:** 10.1016/j.dci.2007.08.005

**Published:** 2008

**Authors:** Anastasia M. Zimmerman, Gene Yeo, Kerstin Howe, Benjamin J. Maddox, Lisa A. Steiner

**Affiliations:** a Department of Biology, Massachusetts Institute of Technology, Cambridge, MA, USA; b Department of Biology, College of Charleston, 66 George Street, Charleston, SC 29424, USA; c Crick-Jacobs Center for Computational and Theoretical Biology, Salk Institute, La Jolla, CA, USA; d Zebrafish Genome Project, Wellcome Trust Sanger Institute, Cambridge, UK

**Keywords:** Immunoglobulin, Zebrafish, Rearrangement, Genome, RSS

## Abstract

In mammals, Immunoglobulin light chain (IgL) are localized to two chromosomal regions (designated *κ* and *λ*). Here we report a genome-wide survey of IgL genes in the zebrafish revealing (V_L_–J_L_–C_L_) clusters spanning 5 separate chromosomes. To elucidate IgL loci present in the zebrafish genome assembly (Zv6), conventional sequence similarity searches and a novel scanning approach based on recombination signal sequence (RSS) motifs were applied. RT-PCR with zebrafish cDNA was used to confirm annotations, evaluate VJ-rearrangement possibilities and show that each chromosomal locus is expressed. In contrast to other vertebrates in which IgL exon usage has been studied, inversional rearrangement between (V_L_–J_L_–C_L_) clusters were found. Inter-cluster rearrangements may convey a selective advantage for editing self-reactive receptors and poise zebrafish by virtue of their extensive numbers of V_L_, J_L_ and C_L_ to have greater potential for immunoglobulin gene shuffling than traditionally studied mice and human models.

## 1 Introduction

The diverse array of immunoglobulins (Ig) and T cell receptors (TCR) are generated from a relatively small number of variable (V), diversity (D), joining (J) and constant region (C) gene segments in the genome. It has been conventional to describe the genomic configurations of these segments as either “translocon” or “multi-clustered” assemblages. The single (V–(D)–J–C) translocon cluster arrangement is typified by mouse and human heavy (IgH) and kappa (*κ*) light (IgL) chain loci where a number of V segments lie upstream of (D_H_), several J and finally one or more C genes.

A departure from a single cluster can be found in the mouse as lambda (*λ*) IgL are arrayed in a 2-cluster (V_2_–(J–C)_2_)–(V–(J–C)_2_) configuration. Because mouse V*_λ_* and J*_λ_* are in the same transcriptional polarity, VJ-rearrangement between the first and second clusters would result in a deletion of intervening V*_λ_* and J*_λ_*, thereby reducing the number of gene segments available for secondary rearrangements. This scenario appears to be avoided as the expressed mouse V*_λ_* repertoire demonstrates a strong bias to rearrange with J*_λ_* within a cluster and rearrangements that leapfrog between clusters appear to be extremely rare [1–3].

Extrapolating from the two *λ* clusters in mice, it has been conventional to broadly define a single Ig “cluster” as any number of V regions upstream of one or more (D), J and C segments [4–6]. To date, the most extensive assemblages of IgH and IgL clusters have been found in cartilaginous fish (sharks and rays) where several hundred (V–(D)–J–C) clusters have been predicted to exist in a single genome [7]. The exact number and arrangement of segments in each cluster, as well as total numbers of clusters are not known. V(D)J-rearrangements in sharks and rays are thought to occur within and not between clusters [5,8]. This within-cluster restriction may be related to the finding that IgH and IgL loci of cartilaginous fishes appear to be in the same transcriptional polarity necessitating that V(D)J-rearrangement is by deletion [9].

Teleost IgL appear to offer a different possibility for VJ-rearrangements. While the IgH segments of bony fish are in a single cluster configuration [10–13], IgL gene segments are multi-clustered [4,14]. Moreover, as V_L_ are often in opposite polarity to J_L_, teleost IgL might have the capacity to undergo inversional VJ-rearrangements both within and between clusters. Rearrangement by inversion, as opposed to deletion, would preserve and invert intervening V_L_, J_L_ and C_L_ thereby maximizing the number of gene segments available for secondary rearrangements. Inversional inter-cluster rearrangements would thus appear to constitute a selective advantage for generating immunoglobulin diversity as gene segments available for secondary rearrangements would be retained while the available exon repertoire for VJ–C combinations would be expanded to include all IgL exons on a given chromosome.

It has long been speculated that inversional inter-cluster IgL rearrangements might be possible in teleosts; however, without a genomic reference sequence such data have remained elusive. The rapidly expanding genomic resources for the zebrafish provide a means by which inter-cluster rearrangement possibilities in an animal harboring extensive germline (V_L_–J_L_–C_L_) clusters can be addressed. In this study, we have combined a suite of bioinformatics-based approaches coupled with EST and cDNA-based cloning strategies to annotate and fit VJ–C transcripts to concordant genomic regions. Collectively, these analyses reveal that inversional VJ-rearrangements occur both within and between IgL clusters in zebrafish. To date, zebrafish represent the only animal model in which inversional rearrangements between IgL clusters have been found.

## 2 Methods

### 2.1 Initial data mining for zebrafish IgL

TBLASTN alignments with V_L_, C_L_, genomic and cDNA sequences from zebrafish, other teleosts, sharks and a variety of mammals were used as queries to scan the zebrafish whole-genome shotgun sequence, trace files, BAC databases, (www.ensembl.org), EST libraries and sequences in NCBI. Identified genes were used in iterative database scans and contigs harboring potential IgL were downloaded from the genome assembly available from The Wellcome Trust Sanger Institute.

### 2.2 RSS identification

RSS flanking V_L_ found by TBLASTN approaches were readily apparent by manual annotation of the sequence immediately downstream of V_L_ segments. Using the EMBOSS [15] package, a pattern search was implemented to find J_L_-specific RSS among the initial genomic contigs found to harbor V_L_ and C_L_. The pattern was a consensus recombination signal sequence (RSS) heptamer and nonamer with a 20–25-base spacer (CACAGTG-N_20–25_-ACAAAAACC) region. Open reading frames flanking identified RSS_36–41_ were scanned for the characteristic amino acid sequence T(X)L(X)V found in J_L_ of sturgeon [16], catfish [17] and zebrafish [18], and classified as J_L_ if this sequence was present.

### 2.3 Genome-wide RSS motif scanning to find zebrafish V_L_ and J_L_

As the zebrafish genome project nears completion, a battery of *ab initio* programs are being used to predict putative exons on a genomic level. We obtained a total of 214,814 Ensembl-predicted zebrafish exons from the Ensembl genome browser [19] (Ensembl Build, V.24a) including 100 bp intronic sequence flanking both sides of each exon. A linear discriminant analysis [20] was then used to score the flanking regions of each exon for the presence or absence of an RSS signal motif.

Based on RSS sequences found by initial data mining, two composite signals, RSS_28_ and RSS_39_, were generated by position weight matrices [21]. Each was a concatenation of 3 ordered signals: a heptamer; a spacer; and a nonamer. A 12-base spacer separates the heptamer and nonamer in RSS_28_ and a 23-base spacer in RSS_39_. Weight matrices consisted of 4 rows (1 for each residue: A, C, G and T) and 1 column for each position tested (*n*=28 or 39). Each matrix entry is a probability *P_x_*(*R*), of a given residue, *R* at a given position *x*, generated from a set of sequences of length *L*. As a control, the background matrix, *B* is defined as *B*(*A*)=0.3, *B*(*C*)=0.2, *B*(*G*)=0.2 and *B*(*T*)=0.3. The log-odds score (**S)** of a given sequence (*s*) of length (*L*) is defined as follows:

$$S_{L}(s)=\underset{x=1:L}{\sum }\log_{2}\;P_{x}(s_{x})-\log_{2}\;B(s_{x})\text{.}$$

Using this formula, sense and antisense strands of each downloaded sequence were scanned for RSS_28_ or RSS_39_. Scores (**S**) were tabulated for each of the 214,844 sequences and a classification function was used to identify putative RSS. Score cutoffs of greater than 6 were used to identify putative heptamer and nonamer signals, and scores greater than 5 were used to discriminate spacers. Exons scored to flank a potential RSS were analyzed for other salient features (invariant residues, leader sequences, folds, framework regions, etc.) consistent with classification as IgL segments.

### 2.4 Annotation of zebrafish IgL

The transcriptional polarity and relative positions of V_L_ and C_L_ in genomic contigs were discerned using the Artemis annotation package. Splice sites between leader and V_L_ exons and J_L_ and C_L_ exons were determined using NNSPLICE and exon boundaries of V_L_, J_L_ and C_L_ were further refined by comparison to known VJ–C cDNA sequences [18].

### 2.5 Zv6 assembly

In the current (Zv6, build August 2006), and previous zebrafish genome assemblies, a number of gaps have been present within the whole-genome shotgun contigs identified to harbor IgL. Gaps circumvent the exact delineation of gene configurations as in subsequent genome builds additional exons may be inserted, thereby reconfiguring the apparent locus. It is also important to note that Zv6 is a draft assembly based on a large number of individuals as source DNA for whole-genome shotgun sequencing (∼500 embryos were pooled). Haplotype variability is known to cause false duplications of loci or contig dropouts in the assembly [22], meaning that precise distances between individual gene segments cannot be discerned based on the whole-genome shotgun sequence alone. To address this, the genome project is sequencing several BAC libraries, with insert sizes ∼110–175 kb, which when complete will constitute several fold coverage of the zebrafish genome.

The zebrafish BAC data currently complement the whole-genome shotgun draft sequence, and as with the human genome, BAC inserts are expected to resolve problems with gaps and haplotypic variability in the assembly. BAC inserts are generally of higher quality than shotgun contigs as a BAC insert is a continuous stretch of DNA from a single individual whereas shotgun contigs are assembled from short (0.5–1.0 kb) overlapping fragments amplified from pooled source DNA. The final zebrafish assembly is projected to consist solely of a BAC-derived sequence with no sequences from the whole-genome shotgun approach (archived information at zebrafish genome project website).

### 2.6 Reference sequences from BAC clones

Given definitive gene orders and accurate physical distances between IgL gene segments are currently restricted to sequences annotated from BAC inserts, we identified a number of BAC clones screened to harbor IgL and had them prioritized for sequencing by the Sanger Institute. To date, 6 such clones have been fully sequenced, 4 of which contain IgL and 2 extend the sequences of BACs zK158E13 and zC276F18 yet do not contain IgL. The IgL annotated from BACs constitute the most amenable germline reference sequences available for evaluating VJ–C rearrangements from cDNA. As such, we have limited our conclusions on adjacent versus distant rearrangements as well as intra- and inter-cluster recombination to VJ–C cDNA clones that can be fitted to IgL segments anchored to fully sequenced BAC clones.

### 2.7 Animals/RNA isolation

Zebrafish (Tübingen) were obtained from the Zebrafish International Resource Center (Eugene, Oregon). RNA was isolated from these fish or their offspring. The zebrafish whole-genome shotgun sequence and BACs sequenced for this study are also of the Tübingen line. Whole zebrafish or organs were frozen in liquid N_2_ and pulverized. RNA was isolated with Trizol (Life Technologies) and reverse-transcribed into cDNA incorporating oligo-dT, random hexamer, or gene-specific primers.

### 2.8 Cloning VJ–C rearrangements from cDNA

Conventional PCR, 3′/5′ FirstChoice RLM-RACE (Ambion) with cDNA templates were used to evaluate IgL exon usage. Reactions were performed using a series of primers optimized to target VJ–C rearranged sequences. In all cases, forward primers were situated in V_L_ regions and reverse primers in C_L_. Amplicons of appropriate sizes were purified from agarose gels using Qiaquick Gel Purification kit (QIAgen), ligated into pCRII-TOPO vectors and transformed into TOP10 cells (Invitrogen). Plasmid DNA was purified using a miniprep kit (QIAgen) and VJ–C clones containing inserts by EcoR1 restriction analysis were sequenced.

### 2.9 Fitting VJ–C cDNA to genomic regions

VJ–C sequences were compared with the V_L_, J_L_ and C_L_ identified in BAC and whole-genome shotgun databases using the Matrix Global Alignment Tool [23]. Clones were assigned to genomic V_L_ contingent upon global alignments exceeding a 95% threshold identity score. This stringent fitting criterion was employed, as the existence of additional IgL segments cannot be ruled out from the current assembly of the zebrafish genome. As the zebrafish genome project is nearing completion and the percent variability in nucleotide sequence of identified V_L_ ranges between 43% and 93%, a 95% criterion is suitably rigorous. Moreover, a 95% threshold exceeds criteria used to fit germline segments to VJ-transcripts in humans [24].

### 2.10 DNA sequencing/sequence data deposition

VJ–C inserts were sequenced bi-directionally on an ABI instrument at the Tufts Medical School Core Facility or the Grice Sequencing Core at the College of Charleston using combinations of T7, SP6 or internal primers. GenBank accession numbers for cloned VJ–C cDNA sequences are as follows: Chr 1 (EF222425, EF222423, EF222424); Chr12 (EF222420, EF222431, EF222434, EF222429, EF222430, EF222433); Chr19 (EF222427, EF222428, EF222426); Chr24 (EF222442, EF222437, EF222441, EF222422, EF222440, EF222421, EF222438, EF222439); Chr25 (EF222432). Accession numbers and corresponding locations of germline V_L_, J_L_ and C_L_ sequences identified from genome shotgun contigs and BAC clones are listed in Table 1.

## 3 Results

### 3.1 A genome-wide IgL annotation spans 5 chromosomes

**Fig. 1:**
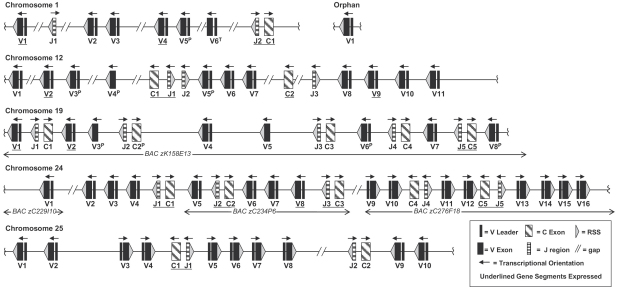
Zebrafish IgL span 5 chromosomes. Overall configurations drawn approximately to scale with exon sizes exaggerated. V^P/T^ designates pseudogene or truncated exon, other notations defined in box. Arrangements are based on Ensembl genome build Zv6 (August 2006). Regions with gaps constitute tentative IgL assemblages as with subsequent genome builds additional exons may be inserted. Where indicated annotation discerned from fully sequenced BAC clone inserts.

A total of 84 IgL gene segments were located in the zebrafish genome assembly Zv6 (Fig. 1). V_L_ were classified functional if they contained leader exons and a downstream RSS. V_L_ and C_L_ were considered pseudogenes if they contained frame shifts or in-frame stop codons. Zebrafish IgL had previously been located to 3 separate chromosomes [25]. Here we provide an extended annotation of zebrafish IgL to include 2 additional chromosomes and considerably more V_L_ and C_L_. With the exception of a single V_L_ (Orphan V1), all 84 IgL gene segments can be anchored to 1 of 5 zebrafish chromosomes. This arrangement in zebrafish is very different from *κ* and *λ* IgL loci of mammals as at least 5 as opposed to 2 chromosomes harbor multiple IgL gene segments including C_L_ regions.

### 3.2 Efficacy of RSS motif scanning

The RSS scan revealed the same contigs to harbor zebrafish IgL as conventional TBLASTN approaches. These results indicate the efficacy of RSS scanning to identify V_L_ or J_L_ from an automatically annotated Ensembl Build and validate 2 independent methods to locate IgL in an emerging genome sequence. Since RSS are more highly conserved than V_L_, the RSS scanning approach may prove especially useful in situations where limited exon coding information is available for use as queries in TBLASTN searches. The RSS approach is also more expedient and represents to our knowledge the first use of a motif signal to comprehensively scan for immunoglobulin segments in a whole-genome context.

### 3.3 Additional genes identified with flanking RSS

The RSS scan in addition to locating V_L_ (with associated RSS_28_) and J_L_ (RSS_39_) revealed numerous V_H_ (RSS_39_) and TCR (RSS_39_) gene segments. Retrieval of V_H_ and TCR sequences was somewhat surprising as the weighted RSS motifs used in our analysis were based on V_L_ (RSS_28_) and J_L_ (RSS_39_) sequences. These findings indicate that RSS scanning is appropriate for surveying emerging genomes for Ig or TCR exons regardless of specific knowledge concerning Ig or TCR coding regions or even lineage-specific RSS motifs. The RSS scan also revealed ortholog of cytochrome C reductase and several immune receptor translocation-associated (IRTA) genes flanked by RSS. Interestingly, IRTA genes have been implicated in translocations into the IgH locus in human B cell malignancies [26], facilitated by an RSS heptamer (CTTAAC) flanking both IRTA and C_H_ regions [27]. The presence of intact RSS flanking IRTA in zebrafish may represent a possible genomic predisposition for Ig translocations involving these genes in a teleost model.

### 3.4 Zebrafish V_L_

**Fig. 2:**
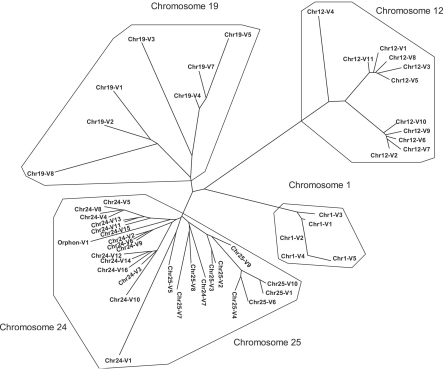
Zebrafish V_L_ group by chromosome. Gene segments aligned in ClustalX and plotted with DrawGram utility of PHYLIP in TreeView.

**Fig. 3:**
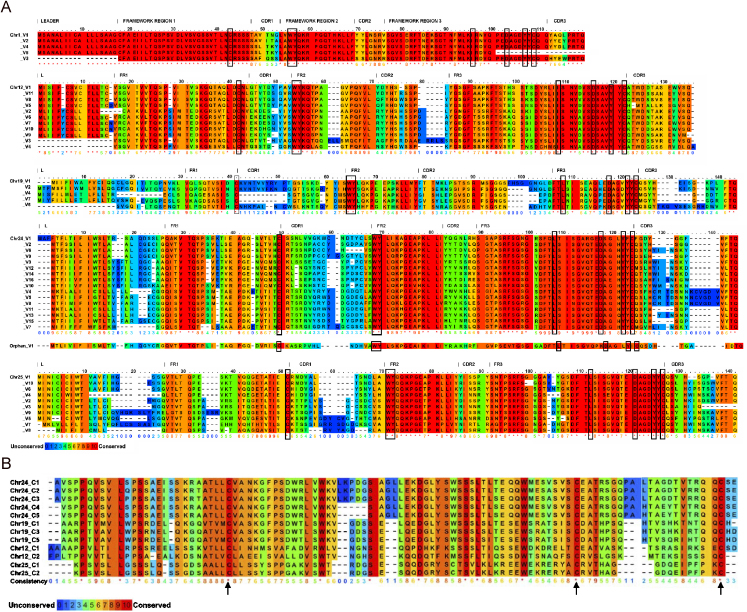
Alignments of inferred amino acid sequences show zebrafish IgL group by chromosome. (A) Alignment of zebrafish V_L_. Conservation (0–10) calculated using PRALINE [29]. Fully conserved positions (score 10) within chromosomes indicated by asterisks and positions invariant among all V_L_ outlined in boxes. Cysteines involved in intra-chain disulfide bridges depicted by arrows on Chr 25. Framework (FR) and complementarity determining regions (CDR) are labeled according to Kabat delineation [30]. (B) Alignment of zebrafish C_L_. Invariant cysteines (indicated by arrows) at residues 28 and 91 are predicted to form intra-chain disulfide bridges whereas cysteine at position 102 is consistent with an inter-chain disulfide bridge with an immunoglobulin heavy chain.

**Fig. 4:**
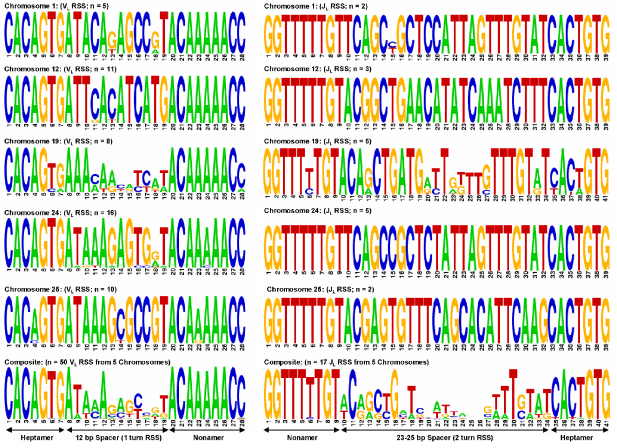
Zebrafish IgL RSS. Sequence logos for V_L_ and J_L_ RSS aligned by chromosome and as composites. Each logo consists of stacks of nucleotides; the overall height of each indicates conservation at that position, while the height of the nucleotides within each stack reflects the relative contribution of each nucleotide to the consensus. Logos constructed using applet available at www.weblogo.berkley.edu and are based on statistical methods previously described [31].

Segments encoding the variable regions of Ig are often grouped by percent identities, with the implication that those most similar descended from a common ancestor [28]. In all but one instance (chr24-V1 vs. chr25-V5), the most similar V_L_ are located on the same chromosome (Fig. 2), suggesting a chromosome-specific pattern of V_L_ evolution with those on chromosomes 24 and 25 having diverged most recently. Zebrafish V_L_ also group by chromosome by percent matrix analysis (Supplementary Fig. 1 online), amino acid alignments (Fig. 3) and RSS logos (Fig. 4). Comparisons of translated V_L_ with sequences in NCBI revealed highest similarities to those of carp, a species phylogenetically close to zebrafish (both species belong to the Cyprinidae family), which is in agreement with previous analyses of V_L_ regions in fish [32].

### 3.5 Zebrafish C_L_

**Fig. 5:**
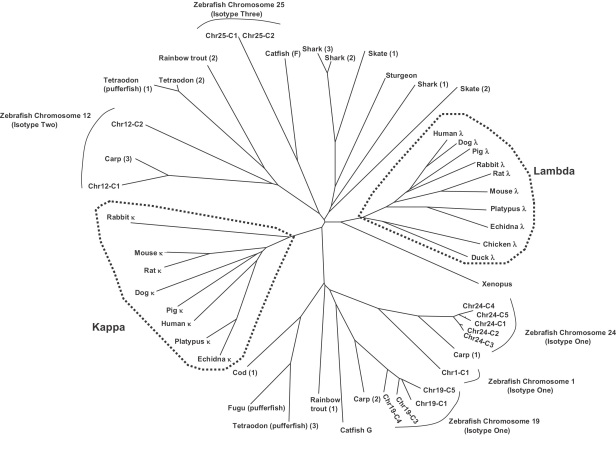
Zebrafish C_L_ are diverged from mammalian IgL. Mammalian kappa (*κ*) and lambda (*λ*) regions are outlined to emphasize the clear divergence of teleost and elasmobranch sequences from traditional IgL classification schemes. Zebrafish C_L_ classified as isotypes designated by Haire et al. [24]. Accession numbers for sequences from GenBank are as follows: Mouse, *Mus musculus*, AC140201, BC080787; Rat, *Rattus norvegicus*, DZ394090, DQ402471; Pig, *Sus domesticus*, M59321, M59322; Human, *Homo sapiens*, NG_000002, BC063599; Dog, *Canis familiaris*, XM_845215, XM_532962; Rabbit, *Oryctolagus cuniculus*, X00231, M25621; Platypus, *Ornithorhynchus anatinus*, AF525122, AF491640; Echidna, *Tachyglossus aculeatus*, AY113112, AF491643; Chicken, *Gallus gallus*, XM_415219; Duck, *Anas platyrhynchos*, X82069; Xenopus, *Xenopus laevis*, BC082892; Skate, *Raja erinacea*, JI9209, L25566; Sandbar shark, *Carcharhinus plumbeus*, U35008, U34992; Horn shark, *Heterodontus francisci*, L25563; Sturgeon, *Acipencer baeri*, X90557; Fugu, *Takifugu rubripes*, AB126061; Tetraodon, *Tetraodon nigroviridis*, BX572609, CR701925, CR720937; Rainbow trout, *Oncorhynchus mykiss*, X68521, AJ231628; Carp, *Cyprinus carpio*, AB035729, AB091120; Crucian carp, *Carassius auratus*, AB201791; Cod, *Gadus morhua*, AF104899; Catfish, *Ictalurus punctatus*, AY165790S2, IPU25704. Alignments were carried out in ClustalW and plotted with DrawGram utility of PHYLIP in TreeView.

Zebrafish C_L_ were compared on a phylogenetic tree to evaluate C_L_ relationships among vertebrates (Fig. 5). This analysis revealed none of the zebrafish C_L_ group with mammalian *λ* or *κ* isotypes. The large phylogenic distances and rapid rates of evolution of antigen receptors appear to preclude a single scheme of IgL classification among vertebrates. Zebrafish C_L_ do however group with C_L_ of other fish and in several cases a common lineage is apparent. For example: zebrafish C_L_ (chr 25) with catfish [33] F; zebrafish C_L_ (chr 19) with catfish G; and C_L_ on chromosomes 24, 1 and 12 group with carp [32] light chain types 1, 2 and 3, respectively (Fig. 5). Collectively, these findings indicate 3 or more C_L_ may have been present in a teleost ancestor and selective pressures have maintained each type in extant species.

### 3.6 VJ–C expression from 5 chromosomes

In total, 23 in-frame (designated as productive) and 3 out-of-frame VJ–C sequences (designated sterile) were cloned. Relationships between these VJ–C clones and their closest match germline segments are shown in Table 2. The upper portion of Table 2 lists clones exceeding 95% threshold criteria for fitting cDNA to germline V_L_. As shown in this table, the C_L_ of clones (EF222427, EF222421, EF222434, EF222432 and EF222433) were fitted in their entirety (100%) to germline segments, suggesting limited polymorphism or somatic mutation in C_L_ among fish of the Tübingen line. Also shown in Table 2, at least one VJ–C clone was fitted to each of the 5 chromosomes depicted in Fig. 1.

The potential to generate IgL from 5 haploid chromosomes presents a conceptually intriguing scenario and implies that if allelic exclusion is to occur in zebrafish, feedback mechanisms are in place to silence a considerable number of IgL segments widely scattered throughout the genome. With functional IgL loci on essentially 10 autosomes, each with multiple V_L_ and J_L_ (zebrafish being diploid and chromosomes 1, 12, 19, 24, 25 do not appear sex-linked [34]), it is plausible that zebrafish have a greater need for gene silencing than *κ* and *λ* systems of mammals.

Although mechanisms underlying allelic exclusion have yet to be fully elucidated in mammals, changes in chromatin, methylation and replication timing are all considered critical to ensure that each B cell can elaborate an antigen receptor of a single type [35]. In mammals, Ig-*κ* positive B cells retain *λ* in a germline configuration [36], whereas Ig-*λ* positive B cells have rearranged Ig-*κ* alleles in addition to rearranged Ig-*λ* allele(s) [37]. These findings imply a hierarchical process starting with *κ*-rearrangement events followed by *λ* if self-reactive or sterile Ig-*κ* receptors are formed.

In Ig-*λ* positive B cells, Ig-*κ* alleles are often inactivated by rearrangements involving the *κ*-deleting element (Kde) in humans [38] or rearranging sequence (RS) in mice [39]. Kde/RS are 3′ to C*κ* and recombine to V*κ* upstream of a rearranged VJ or to an RSS heptamer between J*κ* and C*κ* [40]. Recombination to a J*κ*–C heptamer deletes the C*κ*, while rearrangement to a 5′ V*κ* deletes the entire J*κ*–C*κ* region [41]. As Kde/RS rearrangements render a *κ* locus inoperative, they appear central in *κ*/*λ* isotypic exclusion in mammals.

To see if zebrafish might have Kde/RS elements, we searched zebrafish whole-genome sequence and BAC databases by conventional BLAST approaches, and performed pattern searches of regions 3′ to each C_L_ yet did not find putative Kde/RS homologs. We did find RSS-like heptamers and nonamers (data not shown) within several J_L_–C_L_ intronic regions. It remains to be seen if these RSS are involved in deleting nonproductive VJ-rearrangements or if zebrafish use other means to facilitate allelic exclusion.

### 3.7 VJ-rearrangements in zebrafish

**Fig. 6:**
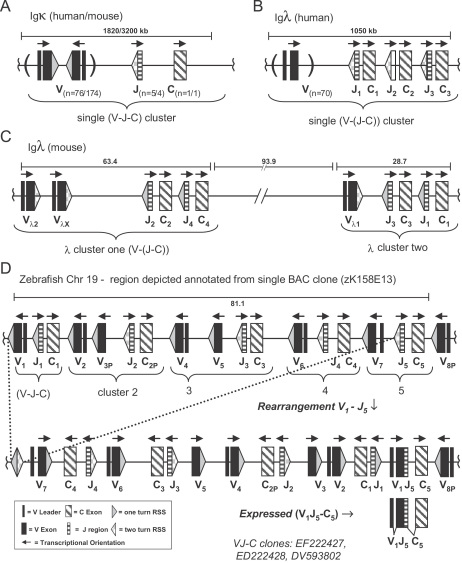
Comparative configuration of IgL loci. (A) Ig*κ* rearrangements are restricted to a single IgL cluster. (B/C) The single transcriptional orientation of *λ* segments of man/mouse necessitates VJ-rearrangement by deletion within and between clusters. (D) Zebrafish have extensive (V–J–C) clusters, those of Chr19 are shown. A potential rearrangement for VJ–C clones (EF222427, EF222428) is depicted. Inversional inter-cluster rearrangements preserve intervening DNA, thus maximizing V_L_ and J_L_ available for subsequent rearrangements. Numbers of segments and physical distances (given in kb) for mouse/human loci are from IMGT [42]. All zebrafish V_L_ and J_L_ identified have 1 and 2 turn RSS similar to Ig*κ* loci of humans/mouse, respectively.

As depicted in Fig. 1, three patterns of transcriptional polarity are evident among zebrafish IgL: V_L_, J_L_ and C_L_ in the same orientation (chr12); V_L_ opposite to J_L_ and C_L_ (chr1,19); and V_L_ in both orientations to J_L_ and C_L_ (chr24, 25). Transcriptional polarities dictate either deletional or inversional rearrangement. For example, given the tentative gene order depicted on chromosome 12, rearrangement between V7 and J1 would result in deletion of (J2, V5^P^, V6). In contrast, an inversional VJ-rearrangement between Chr19-V1 and Chr19-J5 would reposition the intervening gene segments upstream of the rearranged V5–J5 and in opposite transcriptional orientation of the original germline configuration (Fig. 6).

The VJ–C clone (EF222427, Table 2, line 3) is indicative of a VJ-rearrangement between Chr19-V1 and Chr19-J5/C5. This clone (EF222427) was fitted with percent identities of 98.9% and 100% (Table 2), with the next best match being Chr19-V2 (69%) and Chr19-C1 (93%), indicating that assignment of this clone to concordant germline gene segments is sound. Because IgL segments on Chr19 are annotated from a BAC insert (representing an intact section of DNA from a single fish), conclusions concerning distances of the rearrangement can also be made. Of all the VJ–C clones anchored to BACs, this clone represents the most distant recombination as Chr19-V1 and Chr19-J5 are located 81 kb apart (Fig. 6D). This VJ–C clone and others (EF222427, DV593802 and EF222426) show inversional rearrangements that leapfrog C_L_ and as such are indicative of rearrangement between zebrafish IgL clusters.

### 3.8 Inference of selection on V_L_

For VJ–C clones fitted with less than 5% deviation from germline V_L_, assessments of the number of replacement (R) and silent (S) mutations in framework (FR) and complementary determining regions (CDR) were made. The distribution of mutations in corresponding V_L_ regions was analyzed using a multinomial distribution model [43] JAVA applet available at: www.stat.stanford.edu/immunoglobulin. Theoretical probabilities of an excess or scarcity of R and S mutations occurring by chance were computed as accumulation of replacement as opposed to silent mutations in CDRs would indicate antigen selection of variants with improved binding properties [44]. As shown in Table 3, the majority of the V_L_ show statistically significant evidence of selection. These findings indicate CDRs are more plastic, while mutations in FR regions are more likely to be selected against. While these results are not unexpected, they do suggest that V_L_ mutations observed in zebrafish are a product of the antigen-driven somatic hypermutation of Ig loci common in traditionally studied vertebrate animals [45,46].

## 4 Discussion

IgL gene segments have undergone major evolutionary transitions in genomic configurations during vertebrate phylogeny. At one extreme is the chicken, where a single IgL cluster harbors a solitary V_L_ that can undergo primary rearrangement [47,48]. The mouse *λ* locus contains a small number of V_L_ in a (V–V–(J–C)_2_)–(V–(J–C)_2_) configuration, whereas human *κ*, human *λ* and mouse *κ* contain larger numbers of V_L_ in a single discrete cluster per locus (Fig. 6). Herein, we show that zebrafish occupy an entirely different configuration with multiple (V_L_–J_L_–C) clusters arrayed on at least 5 different chromosomes (Fig. 1).

Efforts to evaluate VJ-rearrangements in the context of genomic cluster/exon usage have been largely limited to species for which concordant germline information is available. To date, complete genome-wide annotations of IgL loci are available for only mouse and human [49,50]. Early findings by Southern blotting indicated that the mouse VJ*_λ_* repertoire is strongly biased to VJ-rearrangement within each of the 2 clusters [2,3,51]. Recent sequencing of mouse VJ–C cDNA [52] linked to genomic analyses also indicates that VJ-rearrangement is constrained to a single cluster. Intra-cluster restriction in mice may be due to the large (∼1.75 Mb) distance between the 2 *λ* clusters [42]. Thus, a mouse B cell with a *λ*-rearrangement yielding a self-reactive receptor may be in a potentially dangerous position because of its inability to delete the *λ* rearrangement [53].

In mammals, the potential of generating self-reactive *λ* receptors is abated by timing (*κ* rearrangements occur before *λ*); secondary *κ* rearrangements (facilitated by nested V*_κ_* and J*_κ_*); or unknown mechanisms that limit *λ* expression. The mechanisms underlying the disparate *κ* : *λ* ratio of approximately 10:1 in mice [54] and 3:1 in man [55] remain unresolved. Nevertheless, that a VJ-rearrangement can become fixed constitutes a potential liability as a self-reactive receptor could trigger an autoimmune response. Given that each mouse/human *κ* can sustain a total of 5/4 successive VJ-rearrangements (providing sequential J*_κ_* usage), the probability of a B cell generating a self-reactive *λ* receptor is likely quite small. However, as a *λ* receptor rescues *κ*-deleted B cells from oblivion, there appears an evolutionary tradeoff for sustaining B cells at the expense of generating a final *λ*-rearrangement that cannot be deleted.

Receptor editing (replacing receptors on B cells by continued gene rearrangement) is the principal means by which immature bone marrow B cells become self-tolerant. The potential for receptor editing appears optimized in *κ* as in contrast to *λ* exons, approximately half the V*_κ_* in mouse and human are in opposite transcriptional polarity to J*_κ_*. This flip-flop potential allows *κ* to undergo inversional VJ-rearrangements that preserve the intervening V_L_, J_L_ and associated RSS, between the V*_κ_* and J*_κ_* to be joined. Thus, V_L_ available for a secondary rearrangement is maximized. In the case of mouse and human, inversional VJ-rearrangements precede a single C*_κ_* limiting rearrangement within a single cluster. However, zebrafish with multiple C_L_ on a chromosome are poised to reconfigure an Ig locus by inversional VJ-rearrangements, which place V_L_ from one cluster into another (Fig. 6D). Zebrafish also have more J_L_ (14; haploid) than mouse/man (8/8; haploid) suggesting enhanced potential for IgL receptor editing overall.

Inversional VJ-rearrangements that leapfrog C_L_, as found in zebrafish (Table 2, Fig. 6D), have yet to be documented in any other animal model. While such rearrangements are not possible in mice/humans (each harbor a single C*_κ_* and *λ* loci are limited to deletional rearrangements), it is plausible that inversional VJ-rearrangements between clusters may occur in other animals. For example, rabbits have 2 C*_κ_* isotypes (C*_κ_*_1_ and C*_κ_*_2_) each with its own set of J*_κ_* [56]. This combined with the recent finding of rabbit V*_κ_* are in both transcriptional orientations to J*_κ_* preceding C*_κ_*_1_ [57] may mean that inversional VJ-rearrangements that leapfrog C*_κ_*_1_ are possible. However, it is unknown whether each rabbit C*_κ_* has its own set of V_L_ and efforts to evaluate rearrangements in the context of cluster/exon usage in rabbits have been limited to V_L_ clustered with C*_κ_*_1_ and not the downstream C*_κ_*_2_ [58].

As hundreds of C_L_ have been predicted to exist in cartilaginous fish, it might also appear possible that inter-cluster VJ-rearrangements could also occur in sharks. However, evidence obtained to date suggests that V(D)J rearrangement in cartilaginous fish occurs within and not between clusters [5,8]. Although sharks and teleosts both have multiple clustered IgL loci, differences are evident in the configuration of IgL gene segments in these groups of animals. For example, (V_L_–J_L_–C) clusters are thought to be physically closer to one another in teleosts than in sharks and rays [59]. Additionally, teleost V_L_ are often in opposite polarity to J_L_ and C_L_, whereas IgL segments in cartilaginous fishes are in the same orientation [9]. Thus, inter-cluster rearrangement may be absent in sharks as a result of distance constraints and inversional rearrangement may be lacking as the existence of IgL in the same transcriptional polarity dictates that VJ-recombination is by deletion.

With ongoing efforts to sequence additional genomes it will be interesting to discern whether inversional inter-cluster rearrangements are teleost specific or commonplace in other extant animal lineages. That zebrafish IgL span at least 5 haploid chromosomes with V_L_ and C_L_ grouping by chromosome also supports the notion that gene duplications of IgL loci are a relatively common phenomenon in vertebrate evolution. The finding of appreciably more C_L_ upstream and downstream from arrays of V_L_ and J_L_ (in both transcriptional polarities) in zebrafish raises the possibility that zebrafish B cells may have a greater potential for IgL gene shuffling than traditionally studied mice and human models.

In conclusion, we provide the first evidence of inversional inter-cluster IgL rearrangement in any animal model. This finding and the implication that zebrafish B cells have potential for extensive editing to ablate expression of self-reactive receptors enhances the utility of zebrafish as an emerging immunological model system. In addition, as zebrafish IgL appear to undergo antigen-driven somatic hypermutation, they represent a meaningful branch point in vertebrate phylogeny for further investigations of IgL loci.

Clarifying how allelic exclusion might occur over essentially 10 autosomes in zebrafish may provide considerable insight into elucidating unresolved mechanisms that underlie how B cells elaborate an antigen receptor of a single type while maintaining a genomic reservoir for subsequent diversification.

## Figures and Tables

**Table 1:** Genomic contigs and BAC clones harboring zebrafish IgL

NCBI accession no.	IgL	Location on genomic contigs (Zv6) or BAC clones			Zv4^a^	NCBI accession no.	IgL	Location on genomic contigs (Zv6) or BAC clones			Zv4^a^
		Leader	V_L_, J_L_ or C_L_ exon	RSS				Leader	V_L_, J_L_ or C_L_ exon	RSS	
NW_001511898	Chr1-V1	2175058..2175010	2174933..2174623	2174630..2174603		NW_001512699	Chr24-V1	343628..343702	343769..344100	344093..344120	
	J1	N/A	2179292..2179329	2179253..2179291		NW_001512718	V2	48286..48238	48136..47808	47815..47788	
	V2	2183129..2183081	2182997..2182687	2182694..2182667			V3	54735..54681	54575..54250	54254..54227	
	V3	2186206.. 2186156	2185919..2185609	2185616..2185588			V4	56059..56015	55935..55579	55586..55559	
	V4	2189017..2188969	2188879..2188569	2188576..2188549			J1	N/A	58089..58126	58050..58088	
	V5	2189867..2189819	2189739..2189489	–			C1	N/A	59338..59676	N/A	
	V6	2196118..2196070	2195992..2195887	–		BX001030	V5	1207..1255	763..1117	742..769	*V1a*
	J2	N/A	2197566..2197527	2197526..2197488			J2	N/A	1916..1953	1877..1915	*J1a*
	C1	N/A	2200603..2200922	N/A			C2	N/A	4291..4631	N/A	*C1a*
	Orphan-V1	2207393..2207342	2207268..2206955	2206962..2206935			V6	5916..5964	5489..5814	5469..5496	*V1b*
NW_001510726	Chr12-V1	2043590..2043551	2043472..2043141	2043148..2043121			V7	7166..7199	6640..6983	6620..6647	*V1c*
	V2	2046039..2046002	2045900..2045570	2045577..2045550			V8	9153..9201	8709..9064	8689..8716	*V1d*
	V3	–	2047080..2046721	2046729..2046702			J3	N/A	10012..10049	9973..10011	*J1b*
	V4	–	2048935..2048601	–			C3	N/A	12389..12729	N/A	*C1b*
	C1	N/A	2052109..2051784	N/A	*C2a*	CT573356	V9	50403..50451	49970..50301	49950..49977	*V1k*
	J1	N/A	2053636..2053602	2053675..2053637	*J2a*		V10	48419..48473	47932..48263	47912..47939	
	J2	N/A	2056116..2056082	2056155..2056117	*J2b*		C4	N/A	46427..46752	N/A	
	V5	2058504..2058462	2058386..2058053	2058059..2058032	*V2h*		J4	N/A	45111..45152	45072..45110	
	V6	–	2060582..2060242	2060249..2060222	*V2j*		V11	43805..43853	43406..43731	43386..43413	
	V7	2062657..2062611	2062524..2062185	2062165.. 2062192	*V2c*		V12	42416..42470	41975..42300	41952..41979	
	J3	–	2066768..2066734	2066807..2066769			C5	N/A	38757..39082	N/A	*C1c*
	V8	2067870..2067831	2067752..2067418	2067425..2067398			J5	N/A	37456..37494	37456..37494	*J1c*
	V9	2069527..2069485	2069395..2069061	–			V13	36216..36264	35808..36142	35788..35815	*V1i*
	V10	2071974..2071928	2071841..2071502	2071509..2071482			V14	35040..35094	34599..42300	34576..34603	*V1h*
	V11	2073924..2073885	2073806..2073472	2073479..2073452			V15	33105..33155	32692..33021	32672..32699	*V1g*
NW_001513144	C2	N/A	120680..120365	N/A	*C2b*		V16	31479..31533	31032..31363	31012..31039	*V1f*
BX571825	Chr19-V1	158539..158575	158653..159017	159010..159037	*V1l, V1o*	NW_001512845	Chr25-V1	39800..39752	39665..39355	39359..39332	
	J1	N/A	157776..157813	157814..157852			V2	42256..42228	42108..41778	41785..41758	*V3h*
	C1	N/A	156244..156560	N/A	*C1f*		V3	52525..52553	52674..52982	52995..53024	
	V2	154213..154258	154348..154703	154692..154723	*V1p*		V4	54947..54976	55116..55420	55413..55440	
	V3	–	153237..153560	153579..153606			C1	N/A	57069..56774	N/A	*C3a*
	J2	N/A	152981..153018	153019..153059			J1	N/A	58626..58588	58665..58627	
	C2	N/A	151374.. 151610	N/A			V5	59340..59382	59475..59815	59808..59835	*V3f*
	V4	149032..149080	149250..149525	149518..149545			V6	60934..60982	61093..61418	61411..61438	*V3e*
	V5	136657..136705	137113..137520	137469..137498	*V1r*		V7	61955..62000	62054..62442	62435..62462	*V3d*
	J3	N/A	121221..121260	121261..121299			V8	66844..66816	66783..66464	66473..66444	
	C3	N/A	119649..119968	N/A	*C1h*	NW_001512858	J2	N/A	708..742	669..707	
	V6	86840..86892	86934..87295	87300..87327			C2	N/A	3680..3975	N/A	
	J4	N/A	86400..86437	86438..86477			V9	5961..5933	5848..5484	5491..5464	
	C4	N/A	81554..81769	N/A			V10	9108..9060	8971..8657	8661..8634	
	V7	80568..80616	80750..81051	81044..81071	*V1m, V1t*						
	J5	N/A	80204..80241	80241..80279							
	C5	N/A	77129..77448	N/A	*C1d, C1j*						
	V8	–	72491..72840	72817..72847							

a IgL previously reported [25] are from whole-genome shotgun contigs in Zv4 (September 2004). Zv4 was the first zebrafish genome build to map sequence data to chromosomes and several misalignments were present. IgL on chromosomes 1 and 5 in Zv4 have been reassigned to 24 and 25 in Zv5 (November 2005) and Zv6 (August 2006).

**Table 2:** VJ–C cDNA clones and concordant germ-line gene segments

V(J)–C clone		Most similar germline V_L_				Most similar germline C_L_			Next closest match		ORF^a^
Accession no.	Insert (bp)		% Identity				% Identity				
			V_L_	w/o CDR3	Length^b^		C_L_	Length^b^	V_L_ (%)	C_L_ (%)	
EF222425	647	Chr1-V1	98.7	99.3	308	Chr1-C1	99.3	300	Chr1-V2 (91.3)	Chr24-C5 (78.8)	P
EF222424	626	Chr1-V4	95.9	97.6	297	Chr1-C1	97.9	299	Chr1-V5 (92.6)	Chr24-C5 (78.2)	P
EF222427	713	Chr19-V1	98.9	100	362	Chr19-C5	100	309	Chr19-V2 (69.5)	Chr19-V2 (93.0)	P
EF222428	714	Chr19-V1	99.2	100	362	Chr19-C5	99.0	309	Chr19-V2 (69.8)	Chr19-C3 (94.4)	P
DV593802	750	Chr19-V1	98.6	100	365	Chr19-C5	100	276	Chr19-V2 (69.2)	Chr19-C3 (93.4)	P
EF222426	698	Chr19-V2	98.9	100	353	Chr19-C5	99.3	309	Chr19-V1 (69.5)	Chr24-C3 (94.1)	P
EF222441	574	Chr24-V2	98.8	100	324	Chr24-C1	99.0	195	Chr19-V9 (88.6)	Chr24-C3 (95.8)	P
EF222421	673	Chr24-V8	98.3	100	351	Chr24-C3	100	283	Chr24-V5 (88.5)	Chr24-C2 (99.2)	P
*VJ–C below 95% threshold criteria for fitting germline VL; indicative of somatic mutation, allelic variation or unidentified IgL in genome*											
EF222420	683	Chr12-V9	92.5	95.5	332	Chr12-C1	99.6	324	Chr12-V2 (90.4)	Chr12-C2 (59.3)	P
EF222431	714	Chr12-V9	91.6	95.5	332	Chr12-C1	99.6	324	Chr12-V2 (91.3)	Chr12-C2 (59.3)	P
EF222434	517	Chr12-V5^P^	94.6	97.2	111	Chr12-C1	100	324	Chr12-V11 (91.1)	Chr12-C2 (59.6)	S
EF222429	663	Chr12-V8	89.8	93.1	332	Chr12-C2	99.7	307	Chr12-V11 (89.8)	Chr12-C1 (59.0)	P
EF222430	668	Chr12-V9	94.9	96.6	332	Chr12-C2	97.7	307	Chr12-V6 (92.8)	Chr12-C1 (58.8)	P
EF222433	694	Chr12-V9	92.4	94.9	330	Chr12-C2	100	307	Chr12-V6 (91.6)	Chr12-C1 (59.2)	P
EF222442	318	Chr24-V2	93.7	95.7	80	Chr24-C2	99.5	204	Chr19-V11 (76.9)	Chr24-C3 (98.5)	P
EF222437	582	Chr24-V2	89.2	89.7	324	Chr24-C1	97.4	192	Chr19-V6 (82.7)	Chr24-C3 (92.3)	P
EF222422	688	Chr24-V5	82.2	85.5	305	Chr24-C2	99.0	320	Chr24-V4 (79.1)	Chr24-C3 (98.7)	P
EF222440	341	Chr24-V5	91.0	95.7	110	Chr24-C3	99.0	204	Chr24-V3 (89.2)	Chr24-C2 (98.5)	P
DT318541	666	Chr24-V6	90.4	94.3	318	Chr24-C5	99.6	323	Chr24-V9 (89.7)	Chr24-C4 (99.1)	P
EF222438	326	Chr24-V12	87.4	100	86	Chr24-C1	98.5	204	Chr24-V13 (87.2)	Chr24-C5 (97.6)	S
EF222439	330	Chr24-V7	91.5	93.0	86	Chr24-C3	99.0	230	Chr24-V6 (88.3)	Chr24-C2 (97.1)	S
EF222432	650	Chr25-V9	86.8	90.0	327	Chr25-C1	100	273	Chr25-V2 (87.2)	Chr25-C2 (100)	P

a Single open reading frame=productive (P); lack of ORF=sterile (S).

b Length of global alignments in bp. Sizes of inserts contingent upon primer locations in V_L_ and C_L_, and junctional flexibility.

**Table 3:** Inference of selection on zebrafish V_L_ genes

Clone accession no.	Most similar germline V_L_	FR/CDR	Observed mutations^a^		*P*_M_^b^
			R	S	
EF222425	Chr1-V1	FR	0	1	**0.01236**
		CDR	2	1	0.07169
EF222424	Chr1-V4	FR	3	2	**0.00805**
		CDR	6	1	**0.00373**
EF222420	Chr12-V2	FR	0	2	**0.00003**
		CDR	10	1	**0.00009**
EF222431	Chr12-V2	FR	2	1	**0.00112**
		CDR	12	0	**0.00001**
EF222434	Chr12-V5^P^	FR	0	0	**0.04010**
		CDR	2	1	**0.03863**
EF222430	Chr12-V9	FR	3	3	**0.02144**
		CDR	5	1	0.08865
EF222433	Chr12-V9	FR	4	3	**0.00330**
		CDR	10	3	**0.00614**
EF222427	Chr19-V1	FR	0	0	0.20319
		CDR	1	0	0.08465
DV593802	Chr19-V1	FR	0	0	0.20060
		CDR	2	0	0.08303
EF222426	Chr19-V2	FR	0	0	0.15765
		CDR	2	0	0.05997
EF222442	Chr24-V2	FR	0	0	**0.02731**
		CDR	5	0	**0.00313**
EF222441	Chr24-V2	FR	0	0	**0.03714**
		CDR	1	1	0.81578
EF222440	Chr24-V5	FR	0	0	**0.00408**
		CDR	8	5	**0.00108**
DT318541	Chr24-V6	FR	1	1	**0.00084**
		CDR	8	3	**0.00505**
EF222421	Chr24-V8	FR	0	0	0.13431
		CDR	2	0	**0.04538**

Statistically significant values in bold. P_FR_ is selection to preserve FR and P_CDR_ infers antigen selection of CDR variants.

a Replacement (R) and silent (S) mutations from germline V_L_ (over global alignment lengths reported in Table 1).

b *P*_M_; multinomial probability calculated that excess (for CDR) or scarcity (for FR) of mutations occurred by chance.

## Appendix A Supporting Information

Fig. S1. Comparison of zebrafish V_L_ segments. Amino acids percent identity matrix calculated according to Spalding and Lammers (2004). Identities ⩾70% designation for Ig “V families” (Matsuda, 2004) depicted as shaded boxes. At least 5 distinct families were identified, each by chromosome as shown. Apparent pseudogenes and truncated V_L_ sequences were omitted from analyses.Spalding JB, Lammers (2004) Nucleic Acids Res 32:26–32.Matsuda F (2004) Molecular Biology of B Cells. eds Honjo T, Alt F, Neuberger M (Elsevier) Pp: 1–17.
