# Supplementary material for: Immunoglobulin light chain (IgL) genes in zebrafish: Genomic configurations and inversional rearrangements between (VL–JL–CL) gene clusters
Source: Dev Comp Immunol. 2008;32(4):421–34. doi: 10.1016/j.dci.2007.08.005 (PMC3014032; doi:10.1016/j.dci.2007.08.005)
Supplement: Supplementary file 1 — Fig. S1. Comparison of zebrafish VL segments. Amino acids percent identity matrix calculated according to Spalding and Lammers (2004). Identities ⩾70% designation for Ig “V families” (Matsuda, 2004) depicted as shaded boxes. At least 5 distinct families were identified, each by chromosome as shown. Apparent pseudogenes and truncated VL sequences were omitted from analyses.Spalding JB, Lammers (2004) Nucleic Acids Res 32:26–32.Matsuda F (2004) Molecular Biology of B Cells. eds Honjo T, Alt F, Neuberger M (Elsevier) Pp: 1–17. [file mmc1.pdf]

Supplementary Online Fig. 1: Comparison of zebrafish V_L segments. Amino acids percent identity matrix calculated according to Spalding and Lammers (2004). Identities ≥ 70% designation for Ig "V families" (Matsuda 2004) depicted as shaded boxes. At least 5 distinct families were identified; each by chromosome as shown. Apparent pseudogenes and truncated V_L sequences were omitted from analyses.

Spalding JB, Lammers (2004) *Nucleic Acids Res* 32:26-32.

Matsuda F (2004) *Molecular Biology of B Cells*. eds Honjo T, Alt F, Neuberger M (Elsevier) Pp: 1-17.

	Chr25-V10	Chr25-V9	Chr25-V8	Chr25-V7	Chr25-V6	Chr25-V5	Chr25-V4	Chr25-V3	Chr25-V2	Chr25-V1	Chr24-V16	Chr24-V15	Chr24-V14	Chr24-V13	Chr24-V12	Chr24-V11	Chr24-V10	Chr24-V9	Chr24-V8	Chr24-V7	Chr24-V6	Chr24-V5	Chr24-V4	Chr24-V3	Chr24-V2	Chr24-V1	Chr19-V7	Chr19-V5	Chr19-V4	Chr19-V2	Chr19-V1	Chr12-V11	Chr12-V10	Chr12-V9	Chr12-V8	Chr12-V7	Chr12-V6	Chr12-V5	Chr12-V4	Chr12-V3	Chr12-V2	Chr12-V1	Orphon-V1	Chr1-V5	Chr1-V4	Chr1-V3	Chr1-V2	Chr1-V1
Chr1-V1	36	33	40	30	32	35	34	35	32	36	34	35	41	41	38	40	34	39	30	26	37	33	36	38	38	32	36	27	35	32	26	27	27	26	29	24	25	27	22	21	26	28	31	74	88	70	90	*
Chr1-V2	36	33	41	31	30	38	34	36	32	35	34	36	40	41	35	39	34	39	30	27	38	33	36	39	38	30	41	28	37	32	27	26	25	26	28	24	25	23	22	23	25	26	30	78	92	75	*	
Chr1-V3	36	27	39	30	30	35	36	32	31	32	32	34	33	33	33	31	32	35	26	31	34	30	29	34	33	26	37	34	33	29	25	18	22	23	19	20	22	21	25	23	23	19	25	60	73	*		
Chr1-V4	35	33	40	31	30	34	34	35	31	34	34	35	39	41	35	38	38	40	29	24	36	35	38	38	38	30	37	28	36	32	27	26	27	26	27	25	26	26	23	19	26	28	30	84	*			
Chr1-V5	27	24	30	24	23	26	26	29	24	26	29	28	31	33	30	30	27	31	23	19	27	27	30	31	29	24	28	21	24	24	20	23	23	23	23	21	22	21	13	9	22	24	25	*				
Orphon-V1	46	39	46	40	42	45	40	44	42	45	48	50	47	47	43	48	42	47	45	38	46	47	41	49	46	39	33	29	32	41	35	25	22	23	22	20	24	25	14	20	22	23	*					
Chr12-V1	27	24	29	21	26	27	30	28	30	28	27	25	27	27	26	24	28	24	25	27	26	24	27	31	24	24	24	25	22	26	24	90	54	53	86	58	54	84	51	60	53	*						
Chr12-V2	26	25	25	23	24	24	26	26	28	26	21	29	27	31	25	29	26	27	24	24	27	25	28	27	27	25	21	19	28	23	24	53	84	87	51	87	87	50	39	40	*							
Chr12-V3	23	19	27	16	23	20	25	19	18	16	24	21	25	23	22	24	20	19	23	21	21	23	19	26	18	18	18	27	17	21	21	60	39	38	61	39	38	59	44	*								
Chr12-V4	19	19	21	21	22	26	20	24	26	20	19	25	21	24	21	23	20	23	19	24	24	19	22	21	22	17	23	18	21	23	18	50	38	39	52	38	39	47	*									
Chr12-V5	27	22	27	18	24	28	28	29	29	26	25	28	25	25	24	26	25	24	23	25	28	23	24	29	25	26	23	22	23	25	27	80	48	48	82	50	49	*										
Chr12-V6	24	25	27	22	24	26	27	24	27	25	27	26	30	31	27	28	28	27	23	23	32	24	26	29	28	26	25	20	27	21	24	55	88	89	53	89	*											
Chr12-V7	25	26	26	21	27	25	26	25	27	27	22	22	27	30	24	27	26	26	25	24	27	26	26	25	27	25	23	24	27	20	22	56	91	90	52	*												
Chr12-V8	27	22	27	20	25	27	29	30	28	27	27	26	28	25	25	24	24	25	23	27	26	23	24	27	25	22	26	24	23	27	24	86	52	51	*													
Chr12-V9	25	25	27	22	27	24	27	25	27	26	25	26	30	32	27	28	27	27	24	23	28	25	26	28	29	25	25	20	26	20	25	54	89	*														
Chr12-V10	25	25	26	22	26	25	25	24	26	28	24	23	27	31	23	28	27	27	27	25	28	27	27	27	28	26	25	22	29	21	22	55	*															
Chr12-V11	26	24	29	22	26	26	30	31	28	28	30	27	28	28	26	26	28	27	25	27	26	26	28	30	25	22	23	22	21	26	23	*																
Chr19-V1	45	40	48	39	40	46	46	39	42	43	41	42	44	40	42	39	40	44	39	38	44	39	39	45	44	35	34	32	35	58	*																	
Chr19-V2	49	41	50	38	46	48	48	43	49	47	50	46	50	49	50	45	45	48	43	44	48	44	45	51	47	37	37	37	38	*																		
Chr19-V4	40	40	44	34	37	39	45	41	42	40	38	41	41	39	39	41	41	40	34	42	42	37	40	41	41	37	75	34	*																			
Chr19-V5	40	33	37	29	32	34	33	28	37	31	37	36	34	31	33	34	35	36	32	33	31	37	36	37	33	31	36	*																				
Chr19-V7	41	42	44	37	34	41	42	42	43	42	40	40	41	41	43	40	41	44	34	41	44	36	38	43	46	41	*																					
Chr24-V1	33	36	40	32	34	47	38	40	40	36	40	46	41	39	42	40	38	42	40	39	40	42	36	42	40	*																						
Chr24-V2	51	50	52	49	48	51	52	55	55	54	59	65	62	61	59	60	58	87	57	49	89	59	54	63	*																							
Chr24-V3	54	54	55	52	50	57	56	55	56	50	79	62	85	57	80	54	75	60	52	52	66	56	51	*																								
Chr24-V4	45	41	53	46	47	50	47	45	49	46	48	62	49	74	51	72	47	52	72	46	53	76	*																									
Chr24-V5	50	46	53	45	51	50	48	45	50	51	53	74	56	72	57	74	51	57	84	50	57	*																										
Chr24-V6	51	53	52	50	48	53	55	53	55	51	57	63	63	61	61	56	59	83	53	51	*																											
Chr24-V7	47	48	52	51	45	47	51	48	49	49	47	51	54	52	52	50	46	50	47	*																												
Chr24-V8	45	44	49	43	47	49	45	45	49	48	50	65	53	66	52	66	48	54	*																													
Chr24-V9	51	47	47	46	49	52	51	52	52	52	58	63	59	57	56	60	55	*																														
Chr24-V10	50	50	51	46	47	48	51	45	51	50	69	55	73	50	72	47	*																															
Chr24-V11	45	42	54	46	45	48	45	44	45	49	52	71	55	87	56	*																																
Chr24-V12	49	51	53	47	50	55	54	52	51	49	79	64	84	61	*																																	
Chr24-V13	46	47	59	50	45	50	50	46	49	47	52	72	58	*																																		
Chr24-V14	53	55	54	50	49	54	55	53	54	52	81	58	*																																			
Chr24-V15	52	45	56	44	52	55	49	50	49	54	59	*																																				
Chr24-V16	50	49	48	47	47	51	48	53	52	48	*																																					
Chr25-V1	90	62	59	44	79	60	71	63	64	*																																						
Chr25-V2	66	68	58	47	63	59	60	83	*																																							
Chr25-V3	63	67	57	47	60	60	63	*																																								
Chr25-V4	71	60	65	47	73	60	*																																									
Chr25-V5	59	53	58	46	60	*																																										
Chr25-V6	82	57	54	44	*																																											
Chr25-V7	44	48	52	*																																												
Chr25-V8	58	59	*																																													
Chr25-V9	62	*																																														
Chr25-V10	*																																															

Chromosome 1

Chromosome 12

Chromosome 19

Chromosome 24

Chromosome 25
